# Regional skin wetness perception and its modulation by warm and cold whole body skin temperatures in people with multiple sclerosis

**DOI:** 10.1152/ajpregu.00149.2022

**Published:** 2022-08-29

**Authors:** Aikaterini Christogianni, Richard Bibb, Ashleigh Filtness, Davide Filingeri

**Affiliations:** ^1^Thermosenselab, School of Design and Creative Arts, Loughborough University, Loughborough, United Kingdom; ^2^School of Design and Creative Arts, Loughborough University, Loughborough, United Kingdom; ^3^Thermosenselab, Skin Health Research Group, School of Health Sciences, University of Southampton, Southampton, United Kingdom

**Keywords:** body temperature regulation, multiple sclerosis, skin, thermoreceptors, wetness

## Abstract

Skin wetness sensing is important for thermal stress resilience. Individuals with multiple sclerosis (MS) present greater vulnerability to thermal stress; yet, it is unclear whether they present wetness-sensing abnormalities. We investigated the effects of MS on wetness sensing and their modulation with changes in mean skin temperature (T_sk_). Twelve participants with MS [5 males (M)/7 females (F); 48.3 ± 10.8 yr; Expanded Disability Status Scale (EDSS) range: 1–7] and 11 healthy controls (4 M/7 F; 47.5 ± 11.3 yr) undertook three trials, during which they performed a quantitative sensory test with either a thermoneutral (30.9°C), warm (34.8°C), or cold (26.5°C) mean T_sk_. Participants reported on visual analog scales local wetness perceptions arising from the static and dynamic application of a cold-, neutral-, and warm-wet probe (1.32 cm^2^; water content: 0.8 mL), to the index finger pad, forearm, and forehead. Data were analyzed for the group-level effect of MS, as well as for its individual variability. Our results indicated that MS did not alter skin wetness sensitivity at a group level, across the skin sites and temperature tested, neither under normothermia nor under conditions of shifted thermal state. However, when taking an individualized approach to profiling wetness-sensing abnormalities in MS, we found that 3 of the 12 participants with MS (i.e., 25% of the sample) presented a reduced wetness sensitivity on multiple skin sites and to different wet stimuli (i.e., cold, neutral, and warm wet). We conclude that some individuals with MS may possess reduced wetness sensitivity; however, this sensory symptom may vary greatly at an individual level. Larger-scale studies are warranted to characterize the mechanisms underlying such individual variability.

## INTRODUCTION

Multiple sclerosis (MS) is the most common, autoimmune neurodegenerative disease in young adults, and it is characterized by a plethora of sensory, motor, cognitive and autonomic symptoms (1). There is no cure, and with an increasing 2.8 million people affected worldwide, MS represents a significant public health challenge (2).

Sensory abnormalities such as spontaneous pain, numbness, and thermosensory loss across the body are some of the most common symptoms experienced by people with MS (pwMS) (3–5), and they also represent the first manifestation of the disease in up to 25% of cases (6). “Invisible” MS symptoms such as sensory abnormalities provide a significant burden to the quality of life of pwMS (7), and they can be predictive of disease onset and progression (7–11).

Our research group has recently demonstrated in a group of heat-sensitive pwMS exposed to thermoneutral ambient conditions (*n* = 11) up to 54% of patients presented varying degrees of thermosensory abnormalities in the form of loss in the detection of, and sensitivity to, changes in skin temperature (3). Furthermore, we have previously shown that thermosensory abnormalities can worsen by up to 10% when the body temperature of pwMS increases as a result of heat stress (12), which is in line with a well-known signature of MS pathophysiology, that is, heat-sensitivity or “Uhthoff’s phenomenon” (13).

Promptly sensing when and where across the body we become hot and cold, i.e., thermosensing, is essential to trigger thermal behaviors such as changing clothing insulation, modifying metabolic rates, and drinking, which are necessary to optimally regulate our body temperature and be resilient to heat and cold stress (14, 15). Accordingly, our recent findings have highlighted the possibility that, depending on the degree of thermosensory abnormalities experienced, certain pwMS may be more vulnerable than others to the risks of heat and cold stress (3). However, thermosensing is only one of the two key drivers of human thermal behaviors. Sensing when and where we become wet on the skin due to sweating or contact with a wet surface (e.g., a wet t-shirt), i.e., hygrosensing, plays a fundamental role in the development of human thermal discomfort (16). Importantly, this perception provides a drive for thermal behaviors during heat stress (e.g., active body cooling) that is greater than that provided by changes in skin temperature (17).

Our previous research in healthy young adults has demonstrated that the perception of skin wetness is a phenomenon of the central nervous system, resulting from higher-order neural structures (e.g., somatosensory and insular cortices) optimally integrating multisensory thermal (e.g., cold) and tactile (e.g., stickiness) afferent inputs arising from A-type nerve fibers responding to the skin’s contact with moisture (18, 19). Based on this evidence, it is reasonable to hypothesize that the MS-induced damage to central neural structures is responsible for sensory symptoms (4), including thermosensory loss (3), could also induce skin wetness sensing abnormalities in pwMS.

To date, only one study has evaluated systematically wetness-related sensory abnormalities in a large cohort of ∼800 pwMS (20). In this survey study, we found that around 1 in 3 pwMS experienced wetness on a dry skin site, i.e., phantom wetness and reported it as water trickling on the skin (21).

Although providing initial qualitative evidence on the presence of wetness abnormalities in pwMS, our survey study did not investigate the presence and regional distribution of skin wetness sensitivity abnormalities in response to the application of a controlled wet stimulus to establish the intrinsic wetness sensitivity of the skin in pwMS. As a result, we lack any empirical evidence on the underlying mechanisms for wetness abnormalities in pwMS, such as whether these may result from deficits in the integration of afferent signals within either thermal or tactile peripheral and central neural pathways (19). Furthermore, we have no evidence on whether wetness abnormalities are modulated by changes in whole body mean skin temperature (T_sk_) in pwMS. As both passive heat and cold stress are known triggers of worsening MS symptoms (22), it would be reasonable to hypothesize that wetness abnormalities in pwMS may be exacerbated when whole body mean T_sk_ shifts away from normothermia.

Determining whether MS independently worsens skin wetness sensitivity under normal and shifted thermal states, both in isolation and in combination with thermosensory loss, is important to better understand sensory symptoms in pwMS (7). Furthermore, it could provide the first empirical step in assessing the viability of wetness sensing as a predictive marker of behavioral vulnerability to thermal stress in pwMS. Indeed, should wetness sensing be proven to be deficient in certain pwMS, it could be then investigated whether those pwMS present delayed behavioral responses to changes in whole body mean T_sk_.

The aim of this study was to determine the independent effect of MS, as well as its individual variability, on regional differences in skin wetness perception under normothermic conditions and during changes in whole body mean T_sk_ induced by passive heat and cold stress. We hypothesized that the presence of MS would be associated with a reduction in skin wetness perception across several body regions of pwMS, with this deficit becoming more apparent when both thermal and tactile afferent pathways are engaged, as well as when whole body mean T_sk_ is shifted from thermal neutrality. Furthermore, we evaluated whether certain pwMS may present greater wetness perception abnormalities, owing to the known individual variability in MS presentation and course.

## METHODS

### Ethical Approval

The testing procedures were explained to each participant, and they all gave written informed consent for participation. The study was approved by the Loughborough University Ethics Sub-Committee for Human Participants (no. R17-P094), and testing procedures were in accordance with the tenets of the Declaration of Helsinki (note: the study was not registered in a database). All testing took place at Loughborough (UK) between June 2017 and July 2019, spanning different seasons.

### Participants

Using G*Power 3 software [Heinrich-Heine-Universität Düsseldorf, Germany, Faul et al. (23)], we performed a sample size calculation based on previously published data (24), using an effect size corresponding to a 15 ± 8% (means ± SD) difference in wetness perception between groups. This value is equivalent to ∼1.5 cm on the visual analogue scale (VAS) used for wetness scoring and was considered a meaningful effect size to infer the presence of differences in wetness perception between groups. This choice also aimed to ensure that any group difference would be much greater than any bias introduced by measurement errors. We have estimated that our experimental procedures can carry measurements errors that could potentially induce up to a ∼5% change in perceptual responses (24); hence, adopting an effect size three times greater than that potentially induced by our measurement error was deemed appropriate and sufficient for the purpose of this study. The resulting effect size *f* = .93, combined with an α = 0.05 and a β (power) = 0.8, determined a minimum sample of eight participants per group. Accordingly, we first recruited 12 pwMS [MS group; *n* = 12; 5 males/7 females; age: 48.3 ± 10.8 yr; height: 1.72 ± 0.13 m; body mass: 76.5 ± 17.5 kg; Expanded Disability Status Scale (EDSS) range: 1–7]. Second, we recruited a sex- and age-matched healthy control (CTR) individual for each MS participant but MS participant ID8 (i.e., due to time constrains with study completion). CTR individuals reported no sensory, cardiovascular, neurological, or metabolic diseases (CTR group; *n* = 11; 4 males/7 females; age 47.5 ± 11.3 yr; height: 1.69 ± 0.08 m; body mass: 74.4 ± 16.7 kg).

Participants’ individual characteristics are reported in Table 1. In the MS group, *MS participant 10* reported taking the immunomodulator Copaxone, and the *MS participant 5* reported taking the spasticity medication Baclofen. In addition, *MS participant 5* self-reported commonly experiencing moderate anxiety and pain catastrophizing; *MS participant 6* self-reported commonly experiencing moderate stress, depression and anxiety, and pain catastrophizing; *MS participant 9* self-reported commonly experiencing moderate stress, depression, and anxiety; and MS participant 12 self-reported commonly experiencing moderate anxiety. Three participants with MS (P5, P10, and P12) reported previous experience of abnormal sensitivity to wetness on their skin. Matching MS and CTR groups by age, sex, and (to the extent possible) body dimensions, aimed to minimize confounding factors.

**Table 1.** Individual characteristics of the 12 participants with MS (i.e., MS ID) and 11 healthy CTR tested (i.e., CTR ID)

MS ID	Sex	Age	Height, m	Body Mass, kg	Ethnicity	EDSS (Self-Reported)	MS Type	MS Medications
1	M	38	1.66	69.0	A	1	RR	
2	F	58	1.73	68.9	WE	6.5	RR	
3	F	59	1.6	47.8	WE	6.5	PP	
4	M	44	1.78	76.3	WE	1	RR	
5	F	51	1.57	99.4	WE	6.5	PP	Baclofen
6	M	63	1.97	96.0	WE	6.5	SP	
7	F	33	1.68	104.7	WE	3.5	RR	
8	M	61	1.78	89.5	WE	6	PP	
9	F	53	1.74	61.1	WE	6	PP	
**10**	**M**	**47**	**1.92**	**77.6**	**WE**	**7**	**RR**	**Copaxone**
11	F	33	1.63	63.7	WE	3.5	RR	
**12**	**F**	**40**	**1.61**	**63.6**	**A**	**3**	**RR**	
Mean (SD)		48.3 (10.8)	1.72 (0.13)	76.5 (17.5)		4.7 (2.2)		

CTR ID	Sex	Age, yr	Height, m	Body Mass, kg	Ethnicity
1	M	37	1.77	70.6	WE
2	F	61	1.68	63.2	WE
3	F	60	1.63	76.6	WE
4	M	44	1.78	56.5	WE
5	F	50	1.54	83.8	A
6	M	62	1.70	83.4	WE
7	F	32	1.71	58.4	WE
8	F	55	1.60	66.2	WE
9	M	48	1.82	73.9	WE
10	F	31	1.72	116.7	WE
11	F	42	1.67	68.9	WE
Means (SD)		47.5 (11.3)	1.69 (0.08)	74.4 (16.7)	

Each pwMS but ID 8 had a sex- and age-matched CTR. Boldface rows for the MS ID indicate participants reporting experience of wetness sensing abnormalities. A, Asian; CTR, control; EDSS, Expanded Disability Status Scale; F, female; M, male; MS, multiple sclerosis; PP, primary progressive; pwMS, people with MS; RR, relapsing remitting; SP, secondary progressive; WE, White European.

Exclusion criteria for pwMS were a (self-reported) relapse in the 3 mo before the experiment, to present other neurological diseases, mental illness, sensorimotor disorder, cardiovascular or metabolic disorders, and to be currently taking medications that directly affect cognitive and motor functions.

All participants had lived in the United Kingdom for at least 2 yr before testing, and they had not traveled outside of the United Kingdom for at least 3 mo before testing to minimize differences in acclimatization status. The MS and CTR groups characteristics are presented in Table 1. Participants were instructed to refrain from *1*) performing strenuous exercise in the 48 h preceding testing; *2*) consuming caffeine or alcohol in the 24 h preceding testing; and *3*) consuming food in the 3 h preceding testing.

### Experimental Design

We used a single-blind psychophysical approach based on a well-established quantitative sensory test of skin wetness sensing that we have developed (19, 24), to map individual differences in regional wetness sensitivity between MS and CTR groups undergoing exposures to thermoneutral, hot, and cold environmental conditions.

All participants took part in three experimental sessions on different days (note: time of day between sessions was maintained for each participant) and separated by a minimum of 48 h. During each session participants underwent the same quantitative sensory test during the final 15 min of either one of three 50-min environmental exposures in a climatic chamber, i.e., NEUTRAL (ambient temperature: MS trials 24.0 ± 0.5°C; CTR trials: 24.0 ± 0.5°C; relative humidity MS trials 49.5 ± 0.4%; CTR trials 49.5 ± 0.4%), HEAT, (ambient temperature: MS trials 38.7 ± 2.2°C; CTR trials: 38.9 ± 2.9°C; relative humidity MS trials 51.7 ± 4.5%; CTR trials 47.3 ± 5.4%), or COLD (ambient temperature: MS trials 13.1 ± 1.0°C; CTR trials: 12.6 ± 2.8°C; relative humidity MS trials 48.5 ± 6.3%; CTR trials 44.5 ± 3.0%). The NEUTRAL trial ensured that we could evaluate wetness sensing abnormalities under normothermic conditions. It also provided a control condition against which to evaluate the effects of body heating and cooling during the HEAT and COLD sessions. The HEAT and COLD sessions were designed to induce large changes in whole body mean skin (T_sk_) but not core (rectal) temperature (T_rec_). This was achieved by ramping the ambient temperature in the climatic chamber from a 24°C baseline to a target 38°C (HEAT) or 13°C (COLD) during the 50-min exposure. Pilot trials indicated that 35 min were sufficient to reach the target ambient conditions and induce large changes in mean T_sk_ (i.e., Δ ∼4°C); thereby allowing the delivery of the quantitative sensory test under shifted thermal states during the final 15 min of exposure.

We chose a whole body mean T_sk_ manipulation (as opposed to a core temperature manipulation) to explore the effects of temperature sensitivity in MS (22), as this is a more realistic and relevant scenario encountered by pwMS who may be exposed to sunlight, warm, or cold environments in their daily life. We are aware that the long-standing view on temperature-induced symptoms worsening in MS is that this is driven by temperature-dependent slowing of neural conduction within the central nervous system, due to changes in internal core temperature of as little as ∼0.5°C (13). However, such instances result from increases in core temperature induced by large heat loads, which are likely to be commonly experienced only by pwMS who undergo exercise at significant intensities. In this regard, experimental and epidemiological data indicates that warm ambient-induced increases in mean T_sk_ can decreases postural stability (25) and worsens cognitive status in pwMS (26), thereby supporting a role for changes in mean T_sk_ in worsening MS symptoms.

During the quantitative sensory test, participants had to report the perceived magnitude of local thermal and wetness perceptions arising from the short-duration static (i.e., 5 s) and dynamic (i.e., 5 s) application of a cold-wet (i.e., 25°C), neutral-wet (i.e., 30°C), and warm-wet (i.e., 35°C) handheld, temperature-controllable probe (surface area: 1.32 cm^2^, water content: 0.8 mL). The dynamic application followed immediately the static application and consisted of moving the probe forward by ∼2.5 cm and backward by ∼2.5 cm). Participants reported the magnitude of their local perceptions on two digital visual analog scales for thermal sensation (length 100 mm; anchor points: 0 cold, 100 hot) and wetness perception (length: 100 mm; anchor points: 0 completely dry, 100 completely wet). Perceptions were reported separately for static and dynamic stimulations. As described in our neurophysiological model of skin wetness (19), this approach enabled the differentiation between thermal-only (i.e., static stimulation) and thermotactile (i.e., dynamic stimulation) contributions to skin wetness perception.

We mapped thermal and wetness sensitivity at three different locations over the body; the forehead (over the glabella, between the eyebrows); the ventral side of the left forearm (i.e., mid-distance between elbow and wrist); and the left index finger pad. We chose those body regions because *1*) they include both glabrous (i.e., finger pad) and nonglabrous skin (i.e., forehead and forearm), which we have shown to present differential wetness sensitivity (19) and *2*) they provide a proximal-to-distal organization, which we have shown to impact wetness sensitivity (24).

As per our previous studies (19, 27, 28), all participants were provided with no information on the nature and application of the stimuli to limit expectation biases, and they were only informed about the location of the stimulation. Furthermore, participants underwent a systematic familiarization and calibration to the testing procedures and perceptual scales during a 25-min stabilization period before testing (19, 24). Finally, during all stimuli application, participants were asked to look away from the stimulation site to minimize visual biases. The same investigator performed all testing, to limit any interindividual variability arising from the procedures carried out.

### Experimental Protocol

Participants arrived at the laboratory on testing days and underwent preliminary measurements and preparation. They changed into running shorts (and sport bra) before we assessed their seminude body mass on a precision scale (SECA 874, Germany), and their height on a wall stadiometer. They then self-inserted a rectal thermometer 12 cm beyond the anal sphincter (Viamed, Ltd., West Yorkshire, UK) to record T_rec_ as an indicator of core temperature. Skin thermistors (Grant, Cambridge, UK) were taped to six locations on the left side of the body (cheek, upper chest, outer mid lower arm, hand dorsum, anterior thigh, and lower lateral back) to record local T_sk_ for the estimation of mean T_sk_ according to the following equation (29):

Mean Tsk = (cheek Tsk×0.14) + (upper chest Tsk×0.19) + (outer mid lower arm Tsk×0.11) + (hand dorsum Tsk×0.05) + (anterior tigh Tsk×0.32)+ (lower lateral back Tsk×0.19)

Both T_rec_ and local T_sk_ was recorded at 2 Hz via a dedicated data acquisition system (USB-Temp, MCCdaq) and custom-written software (DASYLab, MCCdaq). We used a washable marker to mark the skin sites to be stimulated, and we gently shaved each site to limit any insulative effect of hair on heat transfer during the application of the stimuli. After this preparation, participants moved into the climatic chamber, which was regulated at ∼24°C (∼50% relative humidity) and underwent 25 min of resting on a chair to adjust to the environmental conditions. During this time, participants were familiarized with the experimental procedures, and calibrated to the visual analogue scales as previously described (24). Also, we marked all stimulation sites with a washable marker to ensure consistency in stimulus delivery. On termination of the 25-min baseline and depending on the experimental sessions, the ambient temperature either remained the same (NEUTRAL) or was ramped to a target 39°C (HEAT), or 13°C (COLD), for the following 50 min. The set ambient temperature was reached within 35 min, at which point the quantitative sensory test commenced. This lasted 15 min and was executed as follow. We first determined the temperature of the first wet stimulus (cold wet, 25°C) and applied a 100% cotton fabric on the handheld thermal probe (surface area: 1.32 cm^2^; NTE-2A, Physitemp), that was then wetted with 0.8 mL of water using a pipette, ensuring full saturation. After a verbal warning (i.e., “I am going to apply the stimulus”), the wet stimulus was applied statically on the participant’s skin for 5 s, during which the participant was encouraged to rate their first thermal and wetness perception. Application pressure was not measured but was controlled to be sufficient to ensure full contact, while not resulting in pronounced skin indention. On acquisition of the perceptual rating, the stimulus was moved dynamically for 5 s forward and backward by ∼2.5 cm, during which the participant was again encouraged to rate their thermal and wetness perception. At this point, we removed the stimulus, gently dried the skin, and then repeated the same procedure for the other stimuli (neutral and warm wet) on the same skin site, before proceeding to the next skin region. The order of testing region was counter-balanced between participants and the order of stimuli (warm vs. neutral vs. cold wet) was counter-balanced between and within participants. Immediately after completion of the quantitative sensory test for all three sites, participants were de-instrumented and exited the climatic chamber.

### Statistical Analysis

All data were assessed for normality of distribution using the Kolmogorov-Smirnov test.

Regarding body temperature data, we averaged mean T_sk_ and T_rec_ values over the final 15-min of each session (i.e., the timeframe coinciding with the delivery of the quantitative sensory test) and then analyzed these data separately for the independent and interactive effects of group (2 levels: MS vs. CTR) and session (3 levels: NEUTRAL, HEAT, COLD), by means of two-way mixed ANOVAs. Post hoc analyses were conducted using Tukey’s test.

To test our first hypothesis on the independent effect of MS on skin wetness sensing, we analyzed wetness perception scores recorded during the NEUTRAL session, separately for each skin site (i.e., finger pad, forearm, forehead) and for each stimulus temperature (i.e., cold-, neutral-, and warm-wet), for the independent and interactive effects of group (2 levels: MS vs. CTR) and mode of stimulation (2 levels: STATIC vs. DYNAMIC), by means of two-way mixed ANOVAs. This approach allowed for the estimation of the independent effect of MS on wetness sensing within each skin site and for each stimulus temperature, under normothermic conditions. Furthermore, we analyzed wetness perception scores resulting from dynamic stimulation during the NEUTRAL, HEAT, and COLD sessions, and collapsed over skin site and stimulus temperature, for the independent and interactive effects of group (2 levels: MS vs. CTR) and experimental session (3 levels: NEUTRAL vs. HEAT vs. COLD), by means of 2-way mixed ANOVAs. This approach allowed for the assessment of the interaction between MS and changes in mean skin temperature on local skin wetness sensing. Identical analyses were conducted using thermal sensations data to establish group differences in temperature sensing. Post hoc analyses were conducted using Sidak’s test.

To test our second hypothesis and evaluate individual abnormalities in wetness sensing in the MS group, we developed individual sensory profiles for each MS participant, and compared these against a large database of normative data from healthy participants, in line with our recent assessment of individual thermosensory abnormalities in pwMS (3). To leverage a larger normative data set than the one provided by the 11 CTR participants tested in the current study, we utilized additional data from healthy participants assessed with identical procedures as the ones described here, during other experiments conducted in our laboratory. These data sets were those presented by Valenza et al. (24) (i.e., *N* = 20; 10 young males and 10 young females) and Wildgoose et al. (30) (*N* = 20; 10 younger and 10 older males), which included assessments of static cold-, neutral-, and warm-wetness sensitivity over the forehead and finger pad. Specifically, on combination of the 3 normative data sets (see Supplemental material; all Supplemental material is available at https://doi.org/10.17028/rd.lboro.16716922.v1), we were able to devise normative values for cold-, neutral-, and warm-wetness sensing based on an *n* = 51 for the forehead, and on an *n* = 31 for the finger pad. The group characteristics of the healthy participants for each normative data set were as follow: forehead (mean age: 36.5 ± 15.3 yr; height: 1.74 ± 0.91 m; body mass: 75.1 ± 14.1 kg; 33% females) and finger pad (mean age: 42.8 ± 16.6 yr; height: 1.76 ± 0.92 m; body mass: 78.6 ± 14.7 kg; 23% females). We note that the normative group body’s mass and height were aligned to those of the MS group (see Table 1), whereas age and proportion of female participants were slightly lower.

Once the normative data sets were established, wetness sensitivity data were calculated individually for each MS patient according to a z-transformation. This transformation enabled the creation of hygrosensory profiles for each individual patient for both a proximal (i.e., forehead) and a distal skin site (i.e., finger pad), and for each stimulus temperature, and for their subsequent assessment against normative data.

This standardized approach is widely used in the context of assessing sensory loss in individual patients. For a detailed overview of the method, see Rolke et al. (31, 32). Analytical procedures used in this study are detailed below.

First, wetness perceptual data (i.e., perceptual magnitude as a 0- to 100-mm value) for each MS and normative participant were log-transformed (Log_10_). Second, log-transformed individual MS perceptual data were z-transformed according to the following equation:

Sensitivity z score =Perceptionindividual MS participant− Mean perceptionCTR groupStandard deviation of mean perceptionCTR group

This transformation results in a sensory profile where perceptual data are presented as standard normal distributions (zero mean, unit variance). Positive *z* scores indicate a gain of function, where the individual with MS is more sensitive to the tested stimuli compared with controls; negative *z* scores indicate a loss of function referring to a lower sensitivity of the individual with MS. Thus, less intense perceptions of either temperature or wetness resulted in negative *z* scores, whereas more intense perceptions resulted in positive z scores. Once the *z* transformation is performed, it is easy to compare individual MS patients’ sensory profiles with the group mean of the normative data. Indeed, the 95% confidence interval of a standard normal distribution is given by the following equation:

$$\mathrm{95\% CI = Mean perceptio}n_{\mathrm{CTR group}}\;\pm 1\mathrm{.96 Standard deviation of mean perceptio}n_{\mathrm{CTR group}}$$

Accordingly, if a sensitivity *z* score for an individual MS patient is greater than +1.96, then the patient exhibits gain of either thermosensory or hygrosensory function (i.e., their reported magnitude of sensation is greater than the 95% CI of the normative group); on the contrary, if a *z* score for an individual MS patient is greater than −1.96, then the patient exhibits loss of thermosensory or hygrosensory function (i.e., their reported magnitude of sensation is smaller than the 95% CI of the normative group).

Data are reported as means, standard deviations (SD), and 95% confidence intervals (CI). Observed power was computed using α = 0.05. Statistical analysis was performed using GraphPad Prism (version 9.0; GraphPad Software, La Jolla, CA). The complete study data set, including the normative data sets, is presented in the Supplemental material.

## RESULTS

### Body Temperature Responses

**Figure 1.**
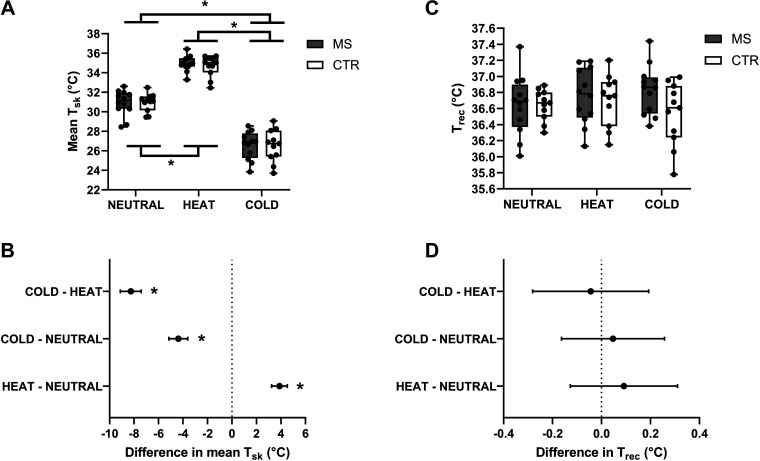
Box and whisker plots of individual and mean (MS, *n* = 12; CTR, *n* = 11) T_sk_ (*A*) and T_rec_ (*B*) data during the NEUTRAL, HEAT, and COLD trials, for both the MS and CTR groups. Mean differences and 95% CI in mean T_sk_ (*C*) and T_rec_ (*D*) among trials are also shown. *P < 0.05. CTR, control; MS, multiple sclerosis; T_rec_, rectal temperature; T_sk_, skin temperature.

MS and CTR participants’ mean T_sk_ changed significantly as a function of the testing session [main effect: *F*_1.8,37.8_ = 357; *P* < 0.0001; 89% of total variation], but not of the group [MS vs. CTR main effect: *F*_1,21_ = 0.07; *P* = 0.783; 0.02% of total variation; Fig. 1*A*]. Post hoc analyses indicated that mean T_sk_ increased largely during the HEAT (mean difference HEAT vs. NEUTRAL: +3.9°C [95% CI: +3.2 to +4.5]; *P* < 0.0001) and decreased largely during the COLD (mean difference COLD vs. NEUTRAL: −4.4°C [95% CI: −5.1 to −3.6]; *P* < 0.0001; Fig. 1*B*).

MS and CTR participants’ T_rec_ did not vary, neither as a function of the testing session [main effect: *F*_1.9,40.6_ = 0.5; *P* = 0.583; 1% of total variation], nor of group [MS vs. CTR main effect: *F*_1,21_ =1.5; *P* = 0.233; 3% of total variation; Fig. 1, *C* and *D*].

These results indicated that our protocol successfully modified mean T_sk_, but not T_rec_, and that MS and CTR groups showed similar body temperature responses to both passive heating and cooling.

### Effect of MS on Wetness Sensing during NEUTRAL: Group-Level Analysis

**Figure 2.**
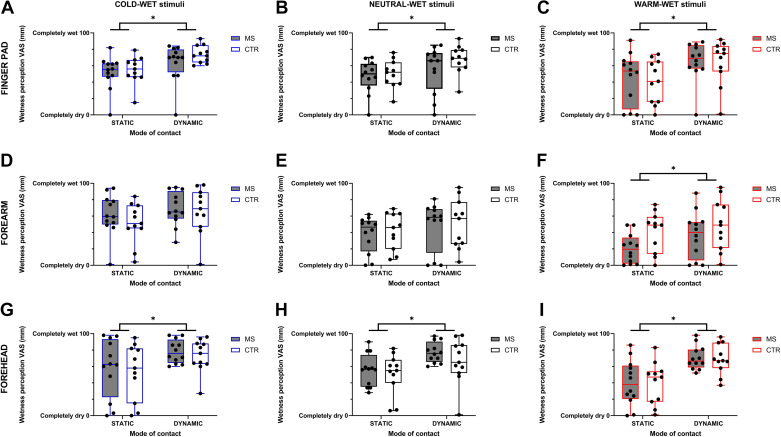
Box and whisker plots of individual and mean (MS, *n* = 12; CTR, *n* = 11) wetness perceptions during static and dynamic applications of the cold-wet, neutral-wet, and warm-wet stimuli to the finger pad (*A*–*C*), forearm (*D*–*F*), and forehead (*G*–*I*), respectively. *Statistically significant differences (*P* < 0.05) for the main effect of mode of contact. CTR, control; MS, multiple sclerosis.

When considering the stimulation of the finger pad, we found no main effect of group (i.e., MS vs. CTR) on wetness perceptions, neither for the cold-wet [*F*_1,21_ = 0.855; *P* = 0.366], neutral-wet [*F*_1,21_ = 0.84; *P* = 0.367], nor warm-wet stimulus [*F*_1,21_ = 0.056; *P* = 0.815]. Yet, we found a significant effect of mode of stimulation on wetness perceptions for the cold-wet [*F*_1,21_ = 26.59; *P* < 0.0001; Fig. 2*A*], neutral-wet [*F*_1,21_ = 17.81; *P* = 0.0004; Fig. 2*B*], and warm-wet stimuli [*F*_1,21_ = 17.76; *P* = 0.0004; Fig. 2*C*]. Specifically, dynamically applied stimuli were perceived as wetter than static ones, during cold (mean difference: 15.5 mm [95% CI: 9.2, 21.7]; corresponding to ∼15% difference), neutral (mean difference: 13.1 mm [95% CI: 6.6, 19.6]; corresponding to ∼13% difference), and warm stimulations (mean difference: 22.1 mm [95% CI: 11.2, 33.0]; corresponding to ∼22% difference).

When considering the stimulation of the forearm, we found no main effect of group (i.e., MS vs. CTR) on wetness perceptions, neither for the cold-wet [*F*_1,21_ = 0.557; *P* = 0.464; Fig. 2*D*], neutral-wet [*F*_1,21_ = 0.104; *P* = 0.749; Fig. 2*E*], nor warm-wet stimulus [*F*_1,21_ = 3.20; *P* = 0.088; Fig. 2*F*]. Yet, we found a significant effect of mode of stimulation on wetness perceptions for the warm-wet stimulus [*F*_1,21_ = 5.05; *P* = 0.035]. Specifically, dynamically applied stimuli were perceived as wetter than static ones, during warm stimulations (mean difference: 12.4 mm [95% CI: 0.9, 23.9]; corresponding to ∼12% difference).

When considering the stimulation of the forehead, we found no main effect of group (i.e., MS vs. CTR) on wetness perceptions, neither for the cold-wet [*F*_1,21_ = 0.284; *P* = 0.599], neutral-wet [*F*_1,21_ = 0.90; *P* = 0.353] nor warm-wet stimulus [*F*_1,21_ = 0.0128; *P* = 0.911]. Yet, we found a significant effect of mode of stimulation on wetness perceptions for the cold-wet [*F*_1,21_ = 10.02; *P* = 0.005; Fig. 2*G*], neutral-wet [*F*_1,21_ = 21.68; *P* = 0.0001; Fig. 2*H*], and warm-wet stimuli [*F*_1,21_ = 28.75; *P* < 0.0001; Fig. 2*I*]. Specifically, dynamically applied stimuli were perceived as wetter than static ones, during cold (mean difference: 19.7 mm [95% CI: 6.7, 32.7]; corresponding to ∼20% difference), neutral (mean difference: 18.3 mm [95% CI: 10.1, 26.5]; corresponding to ∼18% difference), and warm stimulations (mean difference: 29.6 mm [95% CI: 18.2, 41.2]; corresponding to ∼30% difference).

Altogether, the results above indicated that MS did not independently alter skin wetness sensitivity at a group level across all skin sites and temperature tested, and under normothermic conditions. Furthermore, the results indicated that both MS and CTR groups exhibited increased wetness sensitivity on dynamic stimulation of the skin.

### Effect of Whole Body Skin Temperature on Wetness Sensing: Group-Level Analysis

**Figure 3.**
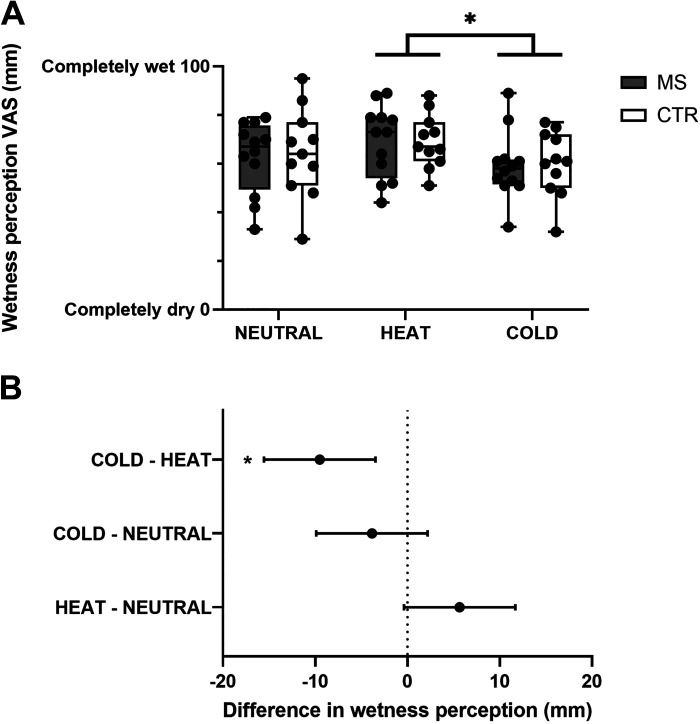
*A*: Box and whisker plots of individual and mean (MS, *n* = 12; CTR, *n* = 11) dynamic wetness perceptions, collapsed over skin sites and stimulus temperature, during NEUTRAL, HEAT, and COLD trials. *B*: mean differences and 95% CI in wetness perceptions among NEUTRAL HEAT, and COLD trials. *P < 0.05. CTR, control; MS, multiple sclerosis.

When considering dynamic wetness perceptions during NEUTRAL, HEAT, and COLD, collapsed over skin site and stimulus temperature, we found no main effect of group [i.e., MS vs. CTR; *F*_1,21_ = 0.0305; *P* = 0.863], yet we found a main effect of experimental session, i.e., change in whole body skin temperature [*F*_2,42_ = 7.76; *P* = 0.001; Fig. 3*A*]. Specifically, we found that, when compared with NEUTRAL, both groups experienced a slight increase in wetness perceptions during HEAT (Fig. 3*B*), as well as a slight decrease in wetness perception during COLD (Fig. 3*B*), to the extent that the difference between HEAT and COLD reached statistical significance (mean difference: 9.5 mm [95% CI 3.4, 15.5]; *P* = 0.001; corresponding to ∼10% difference; Fig. 3*B*).

Altogether, these results indicated that, although air temperature-induced changes in whole body skin temperature modulated skin wetness sensing, this was not independently affected by MS at a group level.

### Individual Analysis of Wetness-Sensing Abnormalities during NEUTRAL

When considering individual sensory profiles (i.e., *z*-scores) for wetness sensing under NEUTRAL conditions for the forehead (Table 2) and finger pad (Table 3), we found that 3 participants with MS (i.e., ID 1, 2, and 10; corresponding to 25% of the MS group), presented abnormal wetness sensing in comparison to our normative data sets. Two of these participants (i.e., ID 1 and 2) presented abnormalities that extended across both the distal and proximal skin site and which involved two thermal qualities (i.e., either warm and cold, or neutral and cold; Table 4), whereas the third participant (i.e., ID 10) presented abnormalities that involved only the distal skin site and two thermal qualities (i.e., warm and cold; Table 4). The abnormal wetness perceptions reported by those three participants corresponded to absolute values of 0 and 3 mm on the 100-mm VAS (see Tables 2 and 3), thereby indicating a loss of sensitivity to wetness.

**Table 2.** Individual absolute wetness perception and z-scores related to the dynamic stimulation of forehead in 12 participants with MS during the NEUTRAL trial

	Forehead					
	Cold-Wet		Neutral-Wet		Warm-Wet	
MS ID	WP, mm	*z*-Score	WP, mm	*z*-Score	WP, mm	*z*-Score
**1**	**0**	−**5.31**	39	0.39	**0**	−**3.28**
**2**	**3**	−**2.26**	59	0.59	46	0.42
3	14	−0.92	28	0.22	26	0.08
4	62	0.39	34	0.32	1	−1.83
5	88	0.70	57	0.58	30	0.16
6	48	0.17	56	0.57	52	0.50
7	95	0.77	90	0.81	86	0.80
8	63	0.41	56	0.57	58	0.56
9	98	0.80	79	0.74	19	−0.11
10	73	0.54	58	0.59	62	0.60
11	60	0.37	34	0.32	24	0.03
12	97	0.79	79	0.74	76	0.72
MS group (*n* = 12) mean WP (SD)	58.4 (35.8)		55.8 (19.7)		40.0 (27.8)	
CTR group (*n* = 51) mean WP (SD)	53.7 (27.4)		40.6 (29.3)		41.3 (28.6)	

Absolute values and *z*-scores are organized per stimulus temperature. Boldface values indicate participants presenting abnormal sensory function (i.e., < −1.96). An *n* = 51 CTR group was used to provide normative data for *z*-score calculations. Absolute wetness perceptions could range from 0 (descriptor: “completely dry”) to 100 mm (descriptor: “completely wet”). CTR, control; MS, multiple sclerosis.

**Table 3.** Individual absolute wetness perception and z-scores related to dynamic stimulation of finger pad in 12 participants with MS during the NEUTRAL trial

	Finger pad					
	Cold-Wet		Neutral-Wet		Warm-Wet	
MS ID	WP, mm	*z*-Score	WP, mm	*z*-Score	WP, mm	*z*-Score
**1**	46	−0.80	44	0.01	**0**	−**3.34**
**2**	62	−0.04	**0**	−**8.52**	1	−1.92
3	55	−0.35	63	0.52	76	0.60
4	65	0.08	70	0.66	52	0.38
5	82	0.67	46	0.08	24	−0.08
6	32	−1.73	69	0.64	63	0.49
7	62	−0.04	33	−0.39	91	0.71
8	56	−0.30	60	0.45	66	0.52
9	48	−0.69	48	0.14	61	0.47
**10**	**0**	−**16.47**	23	−0.89	**0**	−**3.34**
11	51	−0.54	52	0.25	55	0.41
12	63	0.00	55	0.33	49	0.34
MS group (*n* = 12) mean WP (SD)	51.8 (20.4)		46.9 (20.3)		44.8 (31.2)	
CTR group (*n* = 31) mean WP (SD)	67.7 (21.1)		51.7 (24.2)		47.6 (28.6)	

Absolute values and *z*-scores are organized per stimulus temperature. Boldface values indicate participants presenting abnormal sensory function (i.e., < −1.96). An *n* = 31 CTR group was used to provide normative data for *z*-score calculations. Absolute wetness perceptions could range from 0 (descriptor: “completely dry”) to 100 mm (descriptor: “completely wet”). CTR, control; MS, multiple sclerosis.

**Table 4.** Summary for 3 participants with MS presenting abnormal skin wetness sensing

	Forehead			Finger Pad		
MS ID [abnormalities summary]	Cold-Wet	Neutral-Wet	Warm-Wet	Cold-Wet	Neutral-Wet	Warm-Wet
ID 1 [2 skin sites (proximal and distal)/2 thermal qualities]	**X**	X	**X**	X	X	**X**
ID 2 [2 skin sites (proximal and distal)/2 thermal qualities]	**X**	X	X	X	**X**	X
ID 10 [1 skin site (distal)/2 thermal qualities]	**X**	X	X	**X**	X	**X**

Abnormalities are categorized for skin site and stimulus temperature. Boldface X in table body indicate the participant presented an abnormal skin wetness sensory function (i.e., < −1.96). MS, multiple sclerosis.

### Effect of MS on Thermal Sensing during NEUTRAL: Group-Level Analysis

**Figure 4.**
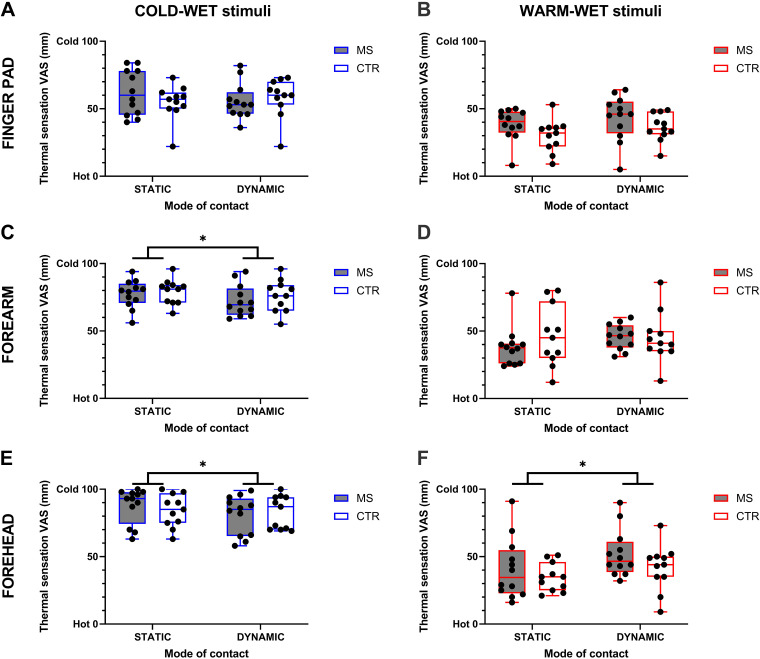
Box and whisker plots of individual and mean (MS, *n* = 12; CTR, *n* = 11) thermal sensations during static and dynamic applications of the cold-wet and warm-wet stimuli to the finger pad (*A* and *B*), forearm (*C* and *D*), and forehead (*E* and *F*), respectively. *Statistically significant differences (*P* < 0.05) for the main effect of mode of contact. CTR, control; MS, multiple sclerosis.

When considering the stimulation of the finger pad, we found no main effect of group (i.e., MS vs. CTR) on thermal sensations, neither for the cold-wet [*F*_1,21_ = 0.165; *P* = 0.688; Fig. 4*A*], nor warm-wet stimulus [*F*_1,21_ = 2.96; *P* = 0.100; Fig. 4*B*]. Similarly, we found no significant effect of mode of stimulation on thermal sensations for the cold-wet [*F*_1,21_ = 0.443; *P* = 0.513] nor warm-wet stimuli [*F*_1,21_ = 3.39; *P* = 0.080].

When considering the stimulation of the forearm, we found no main effect of group (i.e., MS vs. CTR) on thermal sensations, neither for the cold-wet [*F*_1,21_ = 0.449; *P* = 0.510; Fig. 4*C*], nor warm-wet stimulus [*F*_1,21_ = 0.376; *P* = 0.546; Fig. 4*D*]. Yet, we found a significant effect of mode of stimulation on wetness perceptions for the cold-wet stimulus [*F*_1,21_ = 9.41; *P* = 0.006]. Specifically, dynamically applied cold-wet stimuli were perceived as less cold than static ones (mean difference: 4.0 mm [95% CI: 1.3, 6.8]; corresponding to ∼4% difference).

When considering the stimulation of the forehead, we found no main effect of group (i.e., MS vs. CTR) on thermal sensations, neither for the cold-wet [*F*_1,21_ = 0.016; *P* = 0.901; Fig. 4*E*] nor warm-wet stimulus [*F*_1,21_ = 1.45; *P* = 0.241; Fig. 4*F*]. Yet, we found a significant effect of mode of stimulation on wetness perceptions for the cold-wet [*F*_1,21_ = 6.76; *P* = 0.017] and warm-wet stimuli [*F*_1,21_ = 11.36; *P* = 0.003]. Specifically, dynamically applied cold-wet stimuli were perceived as less cold than static ones (mean difference: 4.9 mm [95% CI: 0.8, 7.4]; corresponding to ∼5% difference). Similarly, dynamically applied warm-wet stimuli were perceived as less warm than static ones (mean difference: 9.2 mm [95% CI: 3.5, 14.9]; corresponding to ∼9% difference).

Altogether, the results above indicated that MS did not independently alter skin thermal sensitivity at a group level across all skin sites and temperature tested, and under normothermic conditions. Furthermore, the results indicated that both MS and CTR groups exhibited decreased thermal sensitivity on dynamic stimulation of the skin.

### Effect of Whole Body Skin Temperature on Thermal Sensing: Group-Level Analysis

**Figure 5.**
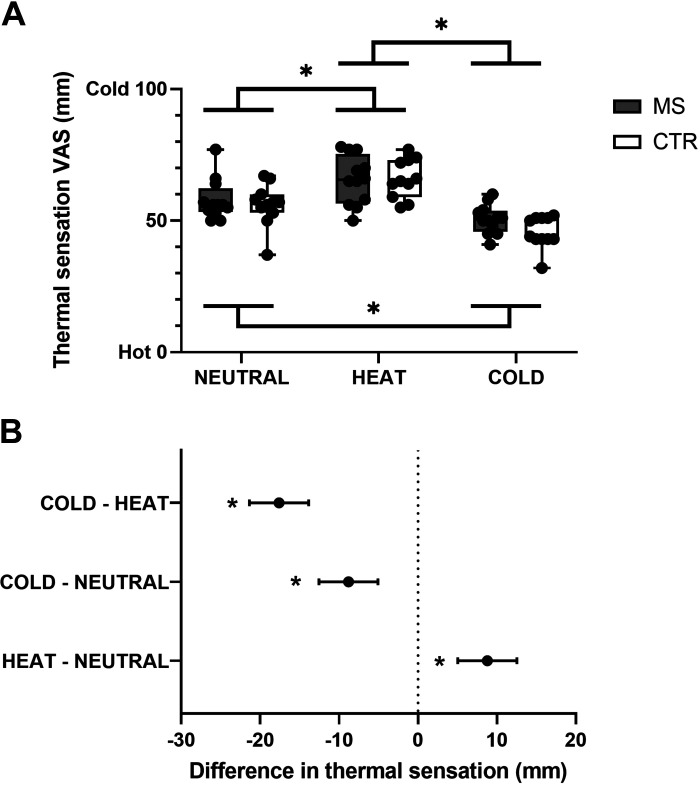
*A*: Box and whisker plots of individual and mean (MS, *n* = 12; CTR, *n* = 11) thermal sensations, collapsed over skin sites, mode of contact, and stimulus temperature, during NEUTRAL, HEAT, and COLD trials. *B*: mean differences and 95% CI in thermal sensations among NEUTRAL HEAT, and COLD trials. *P < 0.05. CTR, control; MS, multiple sclerosis.

When considering thermal sensations during NEUTRAL, HEAT, and COLD, collapsed over skin, stimulus temperature and mode of contact, we found no main effect of group [i.e., MS vs. CTR; *F*_1,21_ = 0.645; *P* = 0.431], yet we found a main effect of experimental session, i.e., change in whole body skin temperature [*F*_2,42_ = 68.18; *P* < 0.0001; Fig. 5*A*]. Specifically, we found that, when compared with NEUTRAL, both groups experienced slightly colder thermal sensations during HEAT (mean difference: 8.8 mm [95% CI 5.0, 12.5]; *P* < 0.0001; corresponding to ∼ 9% difference; Fig. 5*B*), as well as slightly warmer thermal sensations during COLD (mean difference: 8.8 mm [95% CI 5.1, 12.5]; *P* < 0.0001; corresponding to ∼ 9% difference; Fig. 5*B*).

Altogether these results indicated that, although air temperature-induced changes in whole body skin temperature modulated skin temperature sensing, this was not independently affected by MS at a group level.

## DISCUSSION

The aim of this study was to determine the independent effect of MS, as well as its individual variability, on regional sensitivity to skin wetness under normothermic conditions and during changes in whole body mean T_sk_ induced by passive heat and cold stress.

In relation to our first hypothesis, we found that, when taking a group-level approach to the evaluation of the effects of MS, the presence of the disease per se was not associated with a generalized reduction in skin wetness sensitivity across the body of our 13 participants, neither under thermoneutral (see Fig. 2) nor under shifted thermal states (i.e., having a cold or warm whole body mean T_sk_; see Fig. 3). Furthermore, the MS group appeared to use similar multisensory integration mechanisms as the CTR group, whereby dynamically moving wet stimuli were perceived as wetter than static ones, for an equal moisture content (see Fig. 2). Yet, in relation to our second hypothesis, we found that, when taking an individualized approach to wetness sensing abnormalities in MS, 3 of 12 participants with MS (i.e., 25% of our sample) presented a reduced wetness sensitivity that involved multiple skin sites, as well as different wet stimuli (i.e., cold-, neutral-, and warm-wet; see Table 4).

Taken together, our findings indicate that a loss in skin wetness sensitivity may occur in conjunction with the presence of MS; yet this sensory symptom appears to vary greatly at an individual level.

### Group-Level Effects of MS on Skin Wetness Sensing

Contrary to our initial expectations, our group-level analysis did not reveal any overarching effect of MS in modifying skin wetness sensitivity to varying temperature stimuli, and across several skin sites. This is somewhat surprising if one considers that sensory symptoms are some of the most common symptoms experienced by people with MS (pwMS) (3–5). As well as exhibiting an equivalent level of sensitivity to wetness, the MS group demonstrated multisensory integration mechanisms alike the ones we would expect from healthy individuals, whereby a dynamically moving wet stimulus induces greater wetness perceptions than a static wet stimulus for a given moisture level (see Fig. 2) (19, 27, 33). This is because sensing skin wetness relies on the central integration of afferent inputs from both large (i.e., subserving cutaneous mechanoreception via myelinated Aβ fibers) and small peripheral nerve fibers (i.e., subserving cutaneous thermoreception via both nonmyelinated and myelinated C and AΔ fibers) (19).

We know that differences in thermal sensitivity can underlie the independent effect of individual factors such as sex on group-level differences in wetness sensing, whereby greater temperature sensitivity is accompanied by greater wetness sensitivity (24). Our MS group did not present any evident deficit in thermal sensing across the skin sites and temperature stimuli tested (see Fig. 4). Accordingly, it is entirely possible that the lack of group-level thermosensory deficits in our participants with MS would have translated in a lack of group-level differences in wetness sensing. Although thermosensory deficits can be common in MS, they can also be highly variable (3), and the relatively small sample size used in this study may have resulted in an MS group with a low prevalence of thermosensory deficits. Future studies should therefore consider comparing wetness sensitivity levels among larger groups of participants with MS with inherently different levels of thermal sensitivity, to better characterize group-level effects of MS on skin sensitivity.

It is also important to note that group-level wetness sensing in MS changed following increases and decreases in whole body mean T_sk_; yet by an extent no greater than that observed in the CTR group (see Fig. 3). The negative impact of changes in thermal status on symptoms worsening in pwMS has long been known (13); yet we still lack a mechanistic understanding of its pathophysiology, as well as of its effects on nonmotor symptoms, such as sensory deficits (22). Our findings indicate that changes in whole body mean T_sk_ were not sufficient to trigger either a loss of wetness sensing or the exacerbation of an ongoing group-level deficit. The long-standing view on MS heat sensitivity is that this is driven by temperature-dependent slowing of neural conduction within the central nervous system, due to rises in internal core temperature as little as of ∼0.5°C, a notion that was originally derived from in vitro studies of temperature-sensitive demyelinated axons (34). Hence, one could argue that a change in core temperature may have been required to observe any group-level loss in wetness sensing in MS. Yet, there is evidence from Poh et al. (25), who showed that short-term exposures to warm ambient temperatures that elevated skin, but not core temperature, by an extent similar to that induced in the current study (i.e., Δ4°C), triggered a worsening of postural sways in pwMS. This discrepancy may arise from the fact that not all MS individuals are equally affected by changes in thermal status. As such, it could be hypothesized that our MS group may have happened to be overall less affected by thermal stress. A limitation of this study is that we did not conduct trials where both skin and core temperature were concurrently manipulated because of the resulting burden on participants with specific clinical needs. Future studies should therefore consider independent and concurrent manipulation of skin and core temperature to better understand group-level, temperature-induced symptoms worsening in MS.

### Individual Skin Wetness-Sensing Abnormalities in MS

Although a group-level analysis did not reveal overarching effects of MS, our individualized approach to the evaluation of wetness sensing under normothermic conditions uncovered the presence of 3 (out of 12) participants with MS who presented a largely reduced sensitivity to skin wetness across proximal and distal skin sites, and which involved both cold and warm thermal qualities of the wet stimuli we used (see Table 4). This observation indicated that loss in skin wetness sensitivity may occur in conjunction with the presence of MS; yet this sensory symptom clearly varies at an individual level.

The empirical findings of the current study do not provide a population-level estimation of the prevalence of wetness sensory loss in MS assessed via quantitative sensory testing; however, the frequency of this deficit within our tested sample (i.e., 25% of our sample experienced wetness loss) could inform the development of hypotheses for future, large-scale studies of wetness sensing in MS, to determine whether the prevalence rate inferred by the current study aligns with a population-based assessment. Furthermore, these studies may better interrogate the mechanisms underlying such individual variability in skin wetness sensory loss.

When considering the 3 participants with MS who experienced skin wetness sensitivity loss in the current study (i.e., ID 1, 2 and 10), we found that they differed quite broadly in terms of their individual characteristics, i.e., sex (both males and females), age (range: 38–51 yr), ethnicity (both white Europeans and Asians), and EDSS (range: 1–6.5). Yet, we noticed that all 3 participants were affected by Relapsing Remitting MS. The current sample is too limited to allow for meaningful inference on the role of MS type on individual susceptibility to wetness loss. Accordingly, future studies should consider evaluating individual variability in wetness sensing abnormalities in relation to risk factors such as MS type, in larger cohorts of pwMS.

Despite their individual differences, participants ID 1, 2, and 10 presented shared patterns of wetness loss, that is, sensory abnormalities extended across multiple skin sites, and they involved cold, neutral, and warm thermal qualities (see Table 4). Importantly, the fact that loss of sensitivity was repeated within participants (i.e., ID 1 was cold- and warm-wet insensitive on the forehead and warm-wet insensitive on the finger pad; ID2 was cold-wet insensitive on the forehead and neutral-wet insensitive on the finger pad; ID 10 was cold- and warm-wet insensitive on the finger pad) provides evidence that these wetness abnormalities were fairly widespread and consistent. This observation is relevant, as it offers insights on the potential neural pathways involved in MS-induced wetness sensing deficits. Our empirical model of skin wetness proposes that the central integration of wetness sensing relies on afferent thermotactile inputs traveling via the spinothalamic and dorsal-column pathways and centrally integrated by both insular and somatosensory cortices (33). Accordingly, it would be reasonable to hypothesize that those three participants with MS may have presented neural damage to either or both spinal and cortical centers involved in wetness sensing. A major limitation of the present study is that we did not have access to participants’ clinical history with regards to their ongoing MS lesion distribution. Future studies should therefore consider assessing whether such a correlation between nervous system damage (either new or established) and specific types of wetness abnormalities exist, as this approach may provide mechanistic evidence on the nature of those wetness-sensing abnormalities, as well as a noninvasive marker of neural damage in MS.

### Perspectives and Significance

We conclude that a group-level approach to the investigation of MS-induced skin sensory abnormalities could mask the presence of individual skin sensory deficits in this patient group. Our results indicate that MS may be accompanied by a loss in skin wetness sensitivity across the body; yet the occurrence of such a sensory symptom varies greatly at an individual level. This could expose certain pwMS to greater vulnerability to thermal stress, albeit this consideration requires confirmatory empirical evidence. Given the unpredictable location and interindividual variability in MS-induced neurodegeneration within the central nervous system, our findings highlight the need for an individualized, patient-centered profiling of sensory symptoms that concurrently engages multiple neural pathways, such as in the case of skin wetness sensing. Individual factors such as MS type as well as lesion distribution warrant further investigation to determine their role in predisposing certain individuals to a greater risk of skin wetness sensing loss.

## SUPPLEMENTAL DATA

10.17028/rd.lboro.16716922.v1 Supplemental material: https://doi.org/10.17028/rd.lboro.16716922.v1.

## GRANTS

A. C. supported by a PhD Scholarship funded by Loughborough University, UK.

## DISCLOSURES

No conflicts of interest, financial or otherwise, are declared by the authors.

## AUTHOR CONTRIBUTIONS

A.C., R.B., and D.F. conceived and designed research; A.C. performed experiments; A.C., A.F., and D.F. analyzed data; A.C., R.B., A.F., and D.F. interpreted results of experiments; D.F. prepared figures; A.C. and D.F. drafted manuscript; A.C., R.B., A.F., and D.F. edited and revised manuscript; A.C., R.B., A.F., and D.F. approved final version of manuscript.
